# Legionnaires’ disease arising with hirsutism: case report of an extremely confusing event

**DOI:** 10.1186/s12879-021-06247-9

**Published:** 2021-06-06

**Authors:** Gabriella Serio, Francesco Fortarezza, Federica Pezzuto, Luigi Santacroce, Pietro Nazzaro, Emilio Bellitti, Domenica Cavone, Andrea Marzullo, Luigi Vimercati

**Affiliations:** 1 Department of Emergency and Organ Transplantation, Pathology Division, University of Bari, Bari, Italy; 2 Department of Cardiac, Thoracic, Vascular Sciences and Public Health, Pathology Unit, University of Padova, Gabelli St. 61, 35121 Padova, Italy; 3 Department Ionian (DJSGEM), Microbiology and Virology Laboratory, University of Bari, Bari, Italy; 4 Department of Medical Basic Sciences, Neurosciences and Sense Organs, University of Bari, Bari, Italy; 5 Department of Interdisciplinary Medicine, Occupational Health Division, University of Bari, Bari, Italy

**Keywords:** Sepsis, *Legionella pneumophila*, Hirsutism, Autopsy pathology, Case report

## Abstract

**Background:**

Legionella bacteria is a common cause of pneumonia, but the infection may affect several organs in the most serious cases. A systemic involvement ab initio could be non-specific, leading to a diagnostic misinterpretation.

**Case presentation:**

A 33-year-old woman had been complaining of mental confusion, restlessness, aggressiveness, and, subsequently, hirsutism. After 3 weeks, the patient developed pneumonia and died during the hospitalization. The autopsy examination revealed a multi-organ necrotizing exudative disease involving the lung, the heart and the brain. The microbiological tests of tracheal aspirate were positive for *Legionella pneumophila* serotype 1.

**Conclusion:**

The Legionella infection may show a proteiform clinical course and an extra-pulmonary manifestation may be the first sign of the disease. Herein, we report a case of Legionella infection in a young female, presenting with non-specific neurological symptoms and hirsutism at onset, misdiagnosed as a metabolic disease.

## Background

*Legionella pneumophila* infection can pose a diagnostic enigma. The bacterium is a common cause of community-acquired pneumonia (CAP) [1] but the low specificity of the symptoms and the low sensitivity of routine diagnostic tests can delay the diagnosis. The most frequent clinical manifestation of the disease is a form of pneumonia similar to pneumococcal infection. The term of *Legionnaires’ disease* historically describes pneumonia associated with systemic infection due to Legionella bacteria. The disease may variably affect the central nervous system, heart, kidney, gastro-intestinal tract, especially in immunocompromised patients. Rarely, the extra-pulmonary involvement may precede the development of pneumonia or be the only clinical manifestation of the disease, making the diagnosis extremely arduous. Herein, we report a case of Legionella infection affecting a young female with non-specific neurological symptoms and hirsutism at onset, misdiagnosed as a metabolic disease.

## Case presentation

**Fig. 1:**
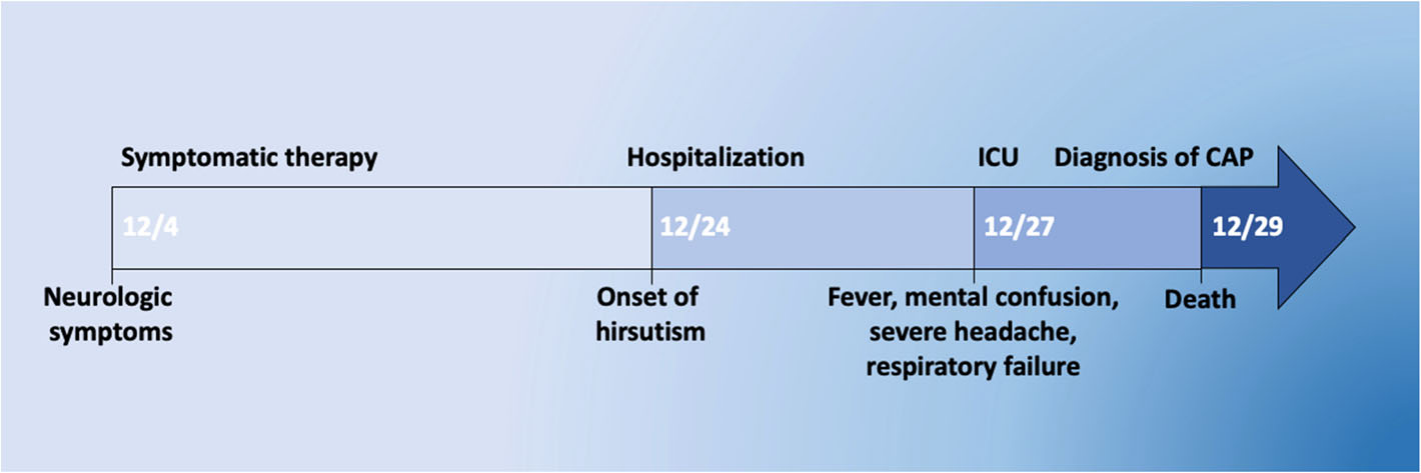
Timeline summarizing the main events in the disease history from its onset (December 4th) until death (December 29th) (ICU: intensive care unit; CAP: community-acquired pneumonia)

**Fig. 2:**
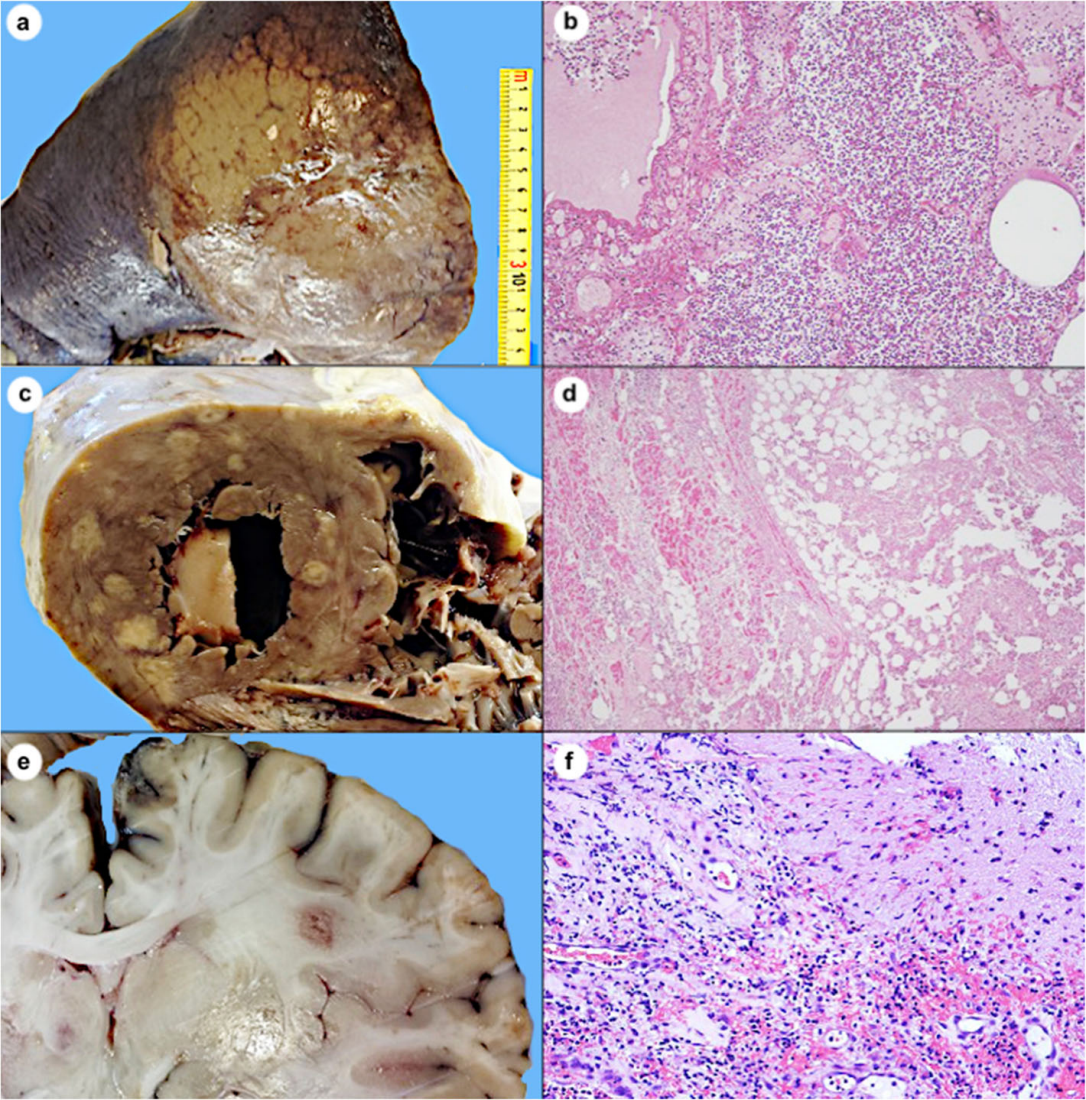
Lung with massive consolidation of the right lower lobe (**a**). Histology shows exudative endo-alveolar and peri-bronchial pneumonia (**b**, Hematoxylin and Eosin, original magnification × 100). The cut surface of the heart shows several necrotic foci and abscesses involving the full thickness of the wall from the serosal surface to the endocardium (**c**). Histology shows neutrophilic myocarditis, evident on right (**d**, Hematoxylin and Eosin, original magnification × 50). Nodular brain mass involving the white matter (**e**) histologically characterized as a polymorphous infiltrate with edema (**f**, Hematoxylin and Eosin, original magnification × 200)

A 33-year-old Rumanian woman was admitted to the Endocrinology Department of Bari University hospital (Italy) for hirsutism. The patient worked as a translator at the police department in her city. The clinical history was unremarkable for endocrinological or gynecological disorders and neoplastic diseases. Twenty days before hospitalization for hirsutism, she had developed mental confusion, restlessness and aggressiveness treated with medical sedatives (Fig. 1). At hospital admission, cardiovascular and respiratory examinations were unremarkable. The chest X-ray was inconsistent for lung disease; the electrocardiogram showed a sinus rhythm and the body temperature was 37.5 °C. Laboratory tests 1 day later demonstrated an increased cortisol level (> 2000 μg) with low adrenocorticotropic hormone (ACTH) (5 ng/L), low albumin 1.5 g/dL, neutrophils 93.3%, platelets count 60 (× 10 + ^3/uL), lactate dehydrogenase 892 U/L, p-fibrinogen 900 mg/dL, P-D-Dimers 4.28 mg/L, hemoglobin 9.8 g/dL, glucose 160 mg/dL, urea 39 mg/dL, calcium 7.9 mmol/dL, ferritin 4449 ng/dL, AST 52 U/L, c-reactive protein 470 mg/L, s-haptoglobin 5.6 g/L, normal sodium levels, procalcitonin 2.9 ng/mL, S-NT-proBNP 10,543 pg/mL, phosphate 2.5 mg/dL; creatinuria 24/h 1118 mg/24 h, glycosuria 24/h 792 mg/24 h, microscopic hematuria. Full body computed tomography-scan showed no masses and tumor markers were negative. Urine culture and rectal swab were negative for common microorganisms. No antibiotic therapy was administered. Three days after hospitalization, owing to a rapid rise in fever, mental confusion, severe headache, and respiratory failure, she was transferred to the intensive care unit (Fig. 1). The laboratory tests were indicative of cardiac distress (S-NT-proBNP 105,797 pg/mL, S-troponin I 24.100 ng/mL cardiac), the chest X-ray showed pneumonia in the right lower lobe and a small quantity of pericardial fluid, consistent with pericarditis. A diagnosis of community-acquired pneumonia with sepsis was made, and broad-spectrum antibiotic therapy was administered pending new microbiological tests, urgently requested on secretions taken from the endotracheal tube and on serum. Unfortunately, the patient died in the meantime. The clinical diagnosis of septic shock was made, and autopsy was performed. The results of the microbiological test by multiplex Polymerase Chain Reaction (PCR) for the detection of Legionella spp., Chlamidia pneumoniae and Mycoplasma pneumoniae in the tracheal aspirate showed positivity for *Legionella pneumophila* serotype 1. Positivity for *Candida albicans* (> 2.000.000) and serum Aspergillus antigen (> 12.70) were also detected. The autopsy revealed multi-organ necrotizing exudative disease involving the lower right lobe (lobar pneumonia) (Fig. 2a), the heart (pancarditis) (Fig. 2c) and the brain, mainly involving the white matter (encephalitis) (Fig. 2e), histologically evident as neutrophilic abscesses (Fig. 2b-d-f). The macroscopic and microscopic examinations of the pituitary gland were negative for tumors. The adrenal glands were diffusely hemorrhagic and the other organs, including ovaries, showed no significant alteration. Results of analyses of water samples carried out in the patient’s apartment showed that the technical measures limit was not exceeded. The search for *Legionella* in the culturable investigations was performed according to guidelines of the multicenter study conducted by the Italian Study Group on Hospital Hygiene (GISIO) of the Italian Society of Hygiene, Preventive Medicine, and Public Health (SItI), Italian Association of Aerobiology (AIA), and the Italian Multidisciplinary Society for the Prevention of Infection in Healthcare Organizations (SIMPIOS) [2].

## Discussion and conclusions

The Legionnaire’s disease is a community-acquired pneumonia caused by the gram-negative bacterium of the Legionella genus, of which there are numerous serogroups which frequently contaminate artificial freshwater systems. Generally, this bacterium affects immunocompromised or debilitated patients. Legionella disease is not always suspected because of its non-specific symptoms and because extra-pulmonary symptoms may precede pneumonia at onset.

The multisystem involvement includes the kidney, liver, heart, gastrointestinal tract. The mechanism involving the nervous system has not yet been well defined and a role of circulating toxins cannot be certainly excluded [3]. Among neurological findings, the most common are mental confusion, headache, irritability and aggressiveness. These clinical manifestations may be underestimated in young patients. In our patient the neurological manifestations, such as mental confusion, restlessness and aggressiveness, were initially treated with symptomatic therapy, without investigating the possible organic causes.

The gold standard for diagnosis of Legionnaires’ disease is culture of patient specimens, but other tests are highly accurate for diagnosing infection, mainly the urinary antigen test and molecular tests, such as PCR and loop-mediated isothermal amplification [4]. Many authors [5, 6] suggested that a close analysis of laboratory tests highlighting high serum levels of ferritin, procalcitonin, C-reactive protein, hyponatremia or low platelet counts, could give rise to the suspicion of a Legionella infection. If microbiology culture test and the molecular tests to identify *Legionella pneumophila* serotypes confirm the diagnosis, targeted antibiotic therapy may be sufficient to aid the patient’s recovery. Unfortunately, a delayed diagnosis and concomitant infections by other pathogens can complicate the clinical picture and an ensuing generalized sepsis may lead to death. The case we describe showed misleading clinical signs and the appearance of hirsutism had raised the suspicion of a metabolic disease. The case is paradigmatic of the “chicken or the egg” situation. The question is whether hypercortisolemia led to immunosuppression responsible for the atypical neurological manifestation of legionellosis or, conversely, if the infection caused the increase in cortisol levels with the related symptoms.

The increase in blood cortisol values can be secondary to exogenous or endogenous causes. Exogenous hypercortisolemia is due to iatrogenic or factitious administration of glucocorticoid drugs. Endogenous forms of hypercortisolemia can be ACTH-dependent or independent. The former underlies a production of ACTH hormone due to tumors, especially adenomas, of the pituitary gland or to ectopic sites of hormone production. ACTH-independent conditions are mainly due to functional tumors of the adrenal glands. More rarely, hypercortisolism is secondary to corticotropin production, micro-macronodular hyperplasia of the adrenal glands. Finally, there are the Pseudo-Cushing syndromes in which hypercortisolemia is not attributable to a dysfunction of the hypothalamus-pituitary-adrenal axis but to other causes, such as estrogenic dysfunctions, alcoholism, major depressive disorders, or else may be idiopathic [7]. The patient’s medical history was negative for the aforementioned conditions (alcoholism, depression, drugs or steroid-based medications, contraceptive therapies, depression). Low ACTH levels ruled out an ACTH-dependent form of hypercortisolism in the first instance. In addition, radiological examinations and the full autopsy did not identify a possible site of hormonal hyperproduction in any organ. We think the cerebral involvement in the form of encephalitis was the first manifestation of the infection, that led to a dysregulation of hypothalamic–pituitary–adrenal axis. The result was an increase in cortisol levels whose clinical manifestation was Cushingoid symptoms (i.e. hirsutism). On the other hand, it should be remembered that sepsis is a stressful condition to which the adrenal gland can respond with a dysregulation of glucocorticoid secretion [8]. Since the septic state occurred about 3 weeks after the neurological symptoms and the onset of hirsutism, we favor the first hypothesis to explain the described unprecedented link between Legionella infection and the endocrinological disorder.

The systemic involvement could have been explained by a systemic vasculitis due to immune mechanisms triggered by Legionella, as reported by other authors [9]. However, the histological examination did not reveal vasculitic lesions, and the multi-organ involvement was shown to be underlying multiple necrotizing exudative abscesses, likely due to the direct action of the pathogen.

A further unusual factor in our case was the detection of normal sodium values. Legionellosis is known to be typically associated with hyponatremia, probably related to an upregulation of vasopressin precursors in severe diseases [10]. In our patient, it could be speculated that the normal sodium levels could be secondary to the mineralocorticoid activity exerted by high cortisol levels.

Our case exemplifies how crucial an accurate analysis of the symptoms and the laboratory tests is to ensure the diagnosis and management of Legionella disease, an emerging community infection, and provide useful diagnostic pointers to reduce “diagnostic fatal error”. Moreover, a prompt diagnosis is important to trace the source of the infection and implement measures to safeguard the health of the general public. Legionella infection may show a proteiform clinical course and an extra-pulmonary manifestation may be the first sign of the disease. To the best of our knowledge, this is the first report of the unusual onset of Legionnaires’ disease in the form of neurological involvement associated with hirsutism, probably an epiphenomenon of the primary development of encephalitis.

## Data Availability

Data sharing is not applicable to this article as no datasets were generated or analyzed during the current study.

## Abbreviations

ACTH: Adrenocorticotropin hormone
CAP: Community-acquired pneumonia
CT: Computed tomography
ICU: Intensive Care Unit
PCR: Polymerase chain reaction
